# SimReg1 is a master switch for biosynthesis and export of simocyclinone D8 and its precursors

**DOI:** 10.1186/2191-0855-2-1

**Published:** 2012-01-03

**Authors:** Liliya Horbal, Yuriy Rebets, Mariya Rabyk, Roman Makitrynskyy, Andriy Luzhetskyy, Victor Fedorenko, Andreas Bechthold

**Affiliations:** 1 Department of Genetics and Biotechnology of Ivan Franko National University of L'viv, Grushevskogo st.4, L'viv 79005, Ukraine; 2 Helmholtz-Institute for Pharmaceutical Research Saarland (HIPS), Helmholtz Center for Infectious Research (HZI), Department Microbial Natural Products Actinobacteria, Metabolic Engineering Group, Saarland University, Campus C2 3 66123 Saarbrücken, Germany; 3 Institut für Pharmazeutische Wissenschaften, Lehrstuhl für Pharmazeutische Biologie und Biotechnologie, Albert-Ludwigs-Universität Freiburg, Stefan-Meier-Strasse 19, 79104 Freiburg, Germany

**Keywords:** Simocyclinone, angucycline, regulation, transport

## Abstract

Analysis of the simocyclinone biosynthesis (*sim*) gene cluster of *Streptomyces antibioticus *Tü6040 led to the identification of a putative pathway specific regulatory gene *simReg1*. *In silico *analysis places the SimReg1 protein in the OmpR-PhoB subfamily of response regulators. Gene replacement of *simReg1 *from the *S. antibioticus *chromosome completely abolishes simocyclinone production indicating that SimReg1 is a key regulator of simocyclinone biosynthesis. Results of the DNA-shift assays and reporter gene expression analysis are consistent with the idea that SimReg1 activates transcription of simocyclinone biosynthesis, transporter genes, regulatory gene *simReg3 *and his own transcription. The presence of extracts (simocyclinone) from *S. antibioticus *Tü6040 *× *pSSimR1-1 could dissociate SimReg1 from promoter regions. A preliminary model for regulation of simocyclinone biosynthesis and export is discussed.

## Introduction

The actinomycetes, including in particular members of the genus *Streptomyces*, are the industrial source for a large number of bioactive compounds employed as antibiotics and other drugs Horinouchi 2007; Bibb and Hesketh 2009. Actinomycetes produce these molecules as part of their ''secondary'' or nonessential metabolism van Wezel et al. 2009. Many *Streptomyces *species are capable of producing more than one secondary metabolite Ohnishi et al. 2008; van Wezel et al. 2009. The timing of the production of secondary metabolites and the amount of the accumulated compounds correlates with the environmental conditions and morphological differentiation van Wezel et al. 2009; Bibb et al. 2009; van Wezel et al. 2011. Furthermore, it has also been associated with the accumulation of small signaling molecules, such as ppGpp, microbial hormones, and late intermediates or end-products of the secondary metabolite biosynthetic pathways Ruiz et al. 2008; O'Rourke et al. 2009; Hsiao et al. 2009; Wang et al. 2009. The influence of all aforementioned factors in most cases is reflected to the activity of the pathway-specific regulatory genes, which are believed to be final checkpoints in the onset of antibiotic production Arias et al. 1999; Nuria et al. 2007; van Wezel et al. 2009; Pulsawat et al. 2007; Wang et al. 2009. Because most antibiotics are potentially lethal to the producing organism, the onset of antibiotic production should be under tight control and mechanisms of self-resistance of producing bacteria must exist. All this requires a precise regulatory network coordinating both, biosynthesis and resistance genes expression Le et al. 2009. That is why very often resistance genes are linked to antibiotic biosynthesis genes Tahlan et al. 2007; Ostash et al. 2008. As our understanding of secondary metabolism advances, it is becoming clear that the relationship between antibiotic production and resistance is more complicated than expected. For example, in *S. coelicolor*, along with the mature antibiotic(s), intermediates of the biosynthetic pathway might activate expression of the export genes, thereby coupling resistance to biosynthesis Hopwood 2007. In *S. cyanogenus *intermediates are able, not only to release repression of the export machinery, but also to de-repress expression of the late biosynthetic enzymes that attach the final sugars to yield mature landomycin A Ostash et al. 2008. However, despite the identification and characterization of numerous genes, which affect antibiotic production and resistance, our understanding of the regulatory networks that govern these processes is far from complete.

**Figure 1:**
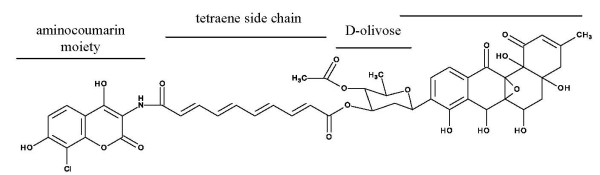
**Structure of simocyclinone D8**.

**Figure 2:**
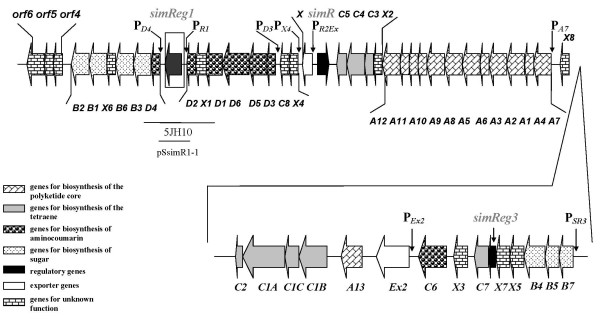
**Schematic representation of the simocyclinone biosynthesis gene cluster (*sim *cluster) of *S. antibioticus *Tü6040**. Fragments used for gene disruption and expression experiments are shown below the genes. Putative promoter regions are indicated with arrows.

A biosynthetic gene cluster usually contains at least one regulatory gene Sheldon et al. 2002; Rebets et al. 2003; Rebets et al. 2008; Chen et al. 2008. This is also the case for the gene cluster of the aminocoumarin antibiotic simocyclinone D8 (Figure 1), produced by *S. antibioticus *Tü6040, that has distinct cytostatic and antibiotic activities Trefzer et al. 2002; Galm et al. 2002; Oppegard et al. 2009; Sadig et al. 2010; Edwards et al. 2009. The simocyclinone biosynthetic gene cluster includes three putative regulatory genes: *simReg1*, *simReg2 *(hereafter *simR*) and *simReg3 *(Figure 2). Recently, the function of SimR was investigated *in vitro *and it was shown to repress the transcription of *simX *gene that encodes simocyclinone efflux pump Le et al. 2009; Le et al. 2011, whereas the function of the two other regulators is still unknown. SimReg1 is the first example of an OmpR-PhoB subfamily regulator, identified in an aminocoumarin biosynthetic gene cluster. Herein, we describe the generation and analysis of the mutant strain deficient in the *simReg1 *gene, mobility shift DNA-binding assays of His-SimReg1 to putative promoter regions and propose a putative model for regulation of the biosynthesis and export of simocyclinones.

## Materials and methods

### Bacterial strains, plasmids, and culture conditions

All strains and plasmids are listed in Table 1. *E. coli *DH5α (Life Technologies) was used for routine subcloning. *E. coli *ET12567 harboring the conjugative plasmid pUB307 (provided by C. P. Smith, UMIST, Manchester, UK) was used to perform intergeneric conjugation from *E. coli *to *Streptomyces *species Flett et al. 1997; Luzhetskyy et al. 2006. For plasmid and total DNA isolation, *E. coli *and *S. antibioticus *strains were grown as described by Sambrook and Russell (2001), and Kieser et al. (2000). For simocyclinone production, *S. antibioticus *strains were grown in liquid NL5 medium (NaCl 1 g l^-1^, KH_2_PO_4 _1 g l^-1^, MgSO4 × 7H_2_O 0.5 g l^-1^, glycerol 25 g l^-1^, L-glutamin - 5.84 g l^-1^, trace elements - 2.0 ml, pH 7.3 prior to sterilization) at 30°C. For conjugation, spores of *S. antibioticus *strains were harvested from a sporulated lawn grown on soya-mannitol or oatmeal medium [Bibr B15][Bibr B19]. When it was necessary, bacterial strains were grown in the presence of respective antibiotics. X-gal and IPTG were used for blue-white colony selection in the case of the pBluescript, pSET152, pKC1139, pKC1218E vectors as described elsewhere Kieser *et al.*, 2000; Sambrook *et al.*, 2001.

**Table 1:** Strains and plasmids

Bacterial strains and plasmids	Description	Source or reference
*E. coli *DH5α	*supE44 ΔlacU169(φ80lacZΔM15)*	Hanahan 1985
*E. coli *BL21 (DE3) pLysS	Host for the heterologous expression of His_6 _-tagged *simReg1*	Novagen
*E. coli *ET12567/pUB307	*hsdR17 recA1endA1gyrA96 thi-1 relA1 dam-13*::Tn9(Cmr) *dcm*-6 *hsd*M; harbors conjugative plasmid pUB307; Cm^r^, Km^r^	Flett et al. 1997
*S. antibioticus *Tü6040	Simocyclinone D8 producing strain	Trefzer et al. 2002
*S. antibioticus*	Derivative of *S. antibioticus *Tü6040 with	This work
ΔsimReg1	disrupted *simReg1 *gene	
*S. antibioticus *ΔsimReg1 × pSSimR1-1	ΔsimReg1 strain carrying plasmid with the intact *simReg1 *gene under its own promoter, used for complementation studies	This work
*S. lividans *1326	Wild type	Hopwood et al. 1985
*S. lividans *× pSimD4script	Derivative of *S. lividans *1326 carrying plasmid with *gusA *gene under the control of the putative promoter of the *simD4 *gene	This work
*S. lividans × *pSimD4script/pUWLsimReg1	Derivative of *S. lividans *1326 carrying plasmid with *gusA *gene under the control of putative promoter of the *simD4 *gene and second plasmid with *simReg1 *gene under the control of P_*ermE*_	This work
*S. lividans *×pGUS	Derivative of *S. lividans *1326 carrying plasmid with promoterless reporter gene *gusA*	This work
*S. lividans *× pGUS/pUWLsimReg1	Derivative of *S. lividans *1326 carrying plasmid with promoterless reporter gene *gusA *and plasmid with *simReg1 *gene under the control of the P_*ermE *_promoter	This work
pBluescriptIIKS ^+^	General purpose cloning vector; Ap^r^	MBI Fermentas
pUC19	General purpose cloning vector; Ap^r^	MBI Fermentas
pSET152	*E. coli/Streptomyces *shuttle vector with φC31 integration system for streptomycetes; Am^r^	Bierman et al. 1992
pKC1218E	pKC1218 derivative expression vector with P_*ermE *_promoter and SCP2* replicon; Am^r^	Ostash et al. 2004
pHYG1	pLitmus38 containing hygromycin resistance cassette *hyg*	C. Olano Univ. de Oviedo, Spain
pKC1139	*E. coli/Streptomyces *shuttle vector with temperature sensitive replicon pSG5, Am^r^	Muth et al. 1989
pUWL-oriT	pUWL-KS derivative harboring *oriT *from pSET152	Zelyas et al. 2009
pET21d	Vector for His-tagged protein expression	Novagen
5JH10	pUC plus *simB3-D4 *segment	Trefzer et al. 2002
pUCsimR1	pUC19 derivative containing *simReg1 *gene	This work
pUCsimR1-hyg	pUCsimR1 derivative with *hyg *cassette cloned into the *simReg1 *coding region	This work
pKCsimR1-hyg	pKC1139 derivative with cloned *simReg1::hyg *construction used for *simReg1 *gene inactivation	This work
pKCEsimR1	pKCE1218 derivative containing *simReg1 *gene under the control of P_*ermE*_	Rebets et al. 2008
pSSimR1	pSET152 plus 2.3 kb *simD4-X1 *segment	This work
pSSimR1-1	pSET152 derivative containing *simReg1 *gene under the control of its own promoter	This work
pMA-simR1	plasmid containing synthetic codon-optimized *simReg1 gene*	Mr. Gene, Heidelberg
pETSR1c-15	pET21d derivative containing synthetic codon-optimized *simReg1 *gene	This work
pGUS	pSET152 derivative containing promoterless reporter gene *gusA*	Myronovskyi et al.2011
pSimD4script	derivative of pGUS harboring *gusA *reporter gene under the promoter of the *simD4 *gene	This work
pUWLsimReg1	derivative of pUWL containing gene *simReg1 *under the control of P_*ermE*_	This work

### DNA manipulations

Isolation of genomic DNA from streptomycetes and plasmid DNA from *E. coli *were carried out using standard protocols Kieser et al. 2000. Restriction enzymes and molecular biology reagents were used according to the recommendation of suppliers (NEB, MBI Fermentas, Promega). DIG DNA labeling and Southern hybridization analyses were performed according to the DIG DNA labeling and detection kit (Roche Applied Science).

### Construction of the plasmid for *simReg1 *inactivation

A 4.3 kb BamHI fragment carrying the entire *simReg1 *gene and its flanking regions was cloned from 5JH10 (Table 1) into pUC19 to yield pUCsimR1 with an unique BsaAI site within the coding region of the *simReg1 *gene. The plasmid pUCsimR1 was digested with BsaAI and ligated to the hygromycin resistance cassette *hyg*, retrieved as an EcoRV fragment from pHYG1 (Table 1). The resulting plasmid pUCsimR1-hyg was digested with BamHI and the fragment containing the *simReg1::hyg *mutant allele was cloned into the shuttle vector pKC1139 to yield pKCsimR1-hyg.

### Generation of the chromosomal mutant *S. antibioticus *ΔsimReg1

The gene disruption plasmid pKCsimR1-hyg was conjugally transferred from *E. coli *into *S. antibioticus *Tü6040. Exconjugants were selected for resistance to apramycin (10 μg ml^-1^). To generate *S. antibioticus *ΔsimReg1 strain, single-crossover mutants were obtained by cultivation of the respective exconjugants at 39°C for 3 days with a further screen for the loss of apramycin resistance as a consequence of a secondary crossover.

### Complementation of the *simReg1 *mutant

The *simReg1 *gene with flanking regions was retrieved from the plasmid pKCEsimR1 Rebets et al. 2008 as a 2.3 kb BamHI fragment and cloned into the BamHI sites of pSET152 to yield pSsimR1. A 1.4 kb SmaI fragment harboring only *simReg1 *with its promoter region was retrieved from pSsimR1 and cloned into EcoRV linearized pSET152 to yield pSsimR1-1.

### Construction of the plasmids for *gusA *reporter fusion system

A 0.5 kb DNA fragment, containing promoter of the *simD4 *gene (P_*D4*_) was amplified from the chromosome *S. antibioticus *Tü6040 using primers simD4_for_script and simD4_rev_script (Table 2). The PCR product was digested with XbaI/KpnI and cloned into the respective sites of pGUS Myronovskyi et al. 2011, giving pSimD4script. In this plasmid transcription of the *gusA *gene is under the control P_*SD4 *_promoter.

**Table 2:** Primers used in this study

Primer	Nucleotide sequence (5'-3')	Purpose	Gene name
SSR1F	ATACCATGGCCCGTGAACGT	SimReg1	*simReg1*
SSR1R	TTTGAATTCATTAATGGTGATGGT	purification	
SR1D4F	TAGAATTCGTGAGCAGATCATGT	DNA-shift assay	P_*D4*_
SR1D4R	TAGAATTCCATTGTGAACCATC		
SD2R1F	TAGAATTCATCGCCACGACCATG	DNA-shift assay	P_*R1*_
SD2R1R	TAGAATTCCGCGGTTCGGCAGA		
simX5D3F	TAGAATTCTGTACAAGGCCTGGT	DNA-shift assay	P_*D3*_
simX5D3R	TAGAATTCGCGACAGGAGCCATA		
simEXX4F	TAGAATTCGACGCCTTCCAGTC	DNA-shift assay	P_*X4*_
simEXX4R	TAGAATTCTCAGAACATCGTCC		
SR2ExXF	AAATCTAGATCAAGCCAGTGCTG	DNA-shift assay	P_*R2Ex*_
SR2ExXR	TTTGAATTCTTGACCACCACTTC		
PSR2F	TCGACGAGGTCCTCTTTG	DNA-shift assay	P_*SR2*_
PSR2R	TCGTATTCATACACCGTAC		
PEx1F	CCAATTGCGCTACGCTCCT	DNA-shift assay	P_*SEx1*_
PEx1R	CCATGTAGGCGGTGACGA		
simA7F	TAAAGCTTCAAAACGGGGTGAAC	DNA-shift assay	P_*A7*_
simA7R	ATAAGCTTGTCGATACCGATCTTC		
PEx2F	ACTTCCCAGAAGTA	DNA-shift assay	P_*Ex2*_
PEx2R	AGAGGGCAGTAGAC		
PR3F	TTTCTAGATGCACCCGATCCTC	DNA-shift assay	P_*SR3*_
PR3R	GAACAGGATTCGCATGAGTACT		
D4For	TATTGGTCGCGCAGTCGTCC	DNA-shift assay	part of the *simD4 *gene
D4Rev	TTGTCCTGCATCATGACGAG		
simD4_for_script	AAATCTAGAGGCGACCGACCCCG GCCGAG	*simD4 *promoter cloning	P_*D4*_
simD4_rev_script	AAAGGTACCCGATCCGGCTGGCA TTACTG		
simReg1_for	AAAAAGCTTTACCTGTATCCAGGGC GGGCACTT	*simReg1 *cloning	*simReg1*
simReg1_ rev	AAAGGATCCGCACAAAGCGGCAGC AATCG		

A 0.8 kb fragment, carrying the *simReg1 *gene, was amplified from the *S. antibioticus *Tü6040 chromosome using the primers simReg1_for and simReg1_rev (Table 2). The amplified DNA fragment was cleaved with HindIII/BamHI and cloned into the respective sites of pUWL-oriT (Table 1), yielding pUWLsimReg1. In this plasmid the *simReg1 *gene is under the control of P_*ermE*_.

### Spectrophotometric measurement of glucuronidase activity in cell lysates

For measurement of GusA activity, mycelium of the *S. lividans *strain harboring both pSimD4script and pUWLsimReg1 plasmids, the control strains *S. lividans *1326 × pSimD4script, *S. lividans *1326 × pGUS, and *S. lividans *1326 × pGUS/pUWLsimReg1 were grown in liquid TSB medium (100 ml) for 2 days at 30°C in a rotary shaker (180 rpm). 1 ml of the pre-culture was inoculated into liquid TSB medium (100 ml) and grown for 5 days at 30°C in a rotary shaker. Mycelium was harvested, washed with distilled water, then resuspended in lysis buffer (50 mM phosphate buffer [pH 7.0], 0.1% Triton X-100, 5 mM DTT, 4 mg ml^-1 ^lysozyme) and incubated for 30 min at 37°C. Lysates were centrifuged for 10 min at 5000 rpm. Then, 0.5 ml of lysate was mixed with 0.5 ml of dilution buffer (50 mM phosphate buffer [pH 7.0], 5 mM DTT, 0.1% Triton X-100) supplemented with 5 μl 0.2 M *p-*nitrophenyl-β-D-glucuronide and used for measuring optical density at λ = 415 nm every minute during 20 min of incubation at 37°C. As a reference, a 1:1 mixture of lysate and dilution buffer was used.

### Analysis of secondary metabolites production

*Streptomyces *strains were grown in liquid TSB medium (50 ml) for 2 days at 30°C in a rotary shaker (180 rpm). Five ml of the pre-cultures were inoculated into liquid NL5 medium (100 ml) and the cultures were grown for 5 days at 30°C in a rotary shaker. The culture broths were extracted three times with 100 ml of ethyl acetate. The extracts were dried in vacuum and dissolved in methanol (200-400 μl). The metabolites were analyzed by high-pressure liquid chromatography-mass spectrometry (HPLC-MS) Schimana et al. 2001. 10 ml of each culture were taken and lyophilized. The dry weight of each sample was measured. In all cases amounts of antibiotic were referred back to equal amounts of biomass (dry weight) and are mean values from at least three independent experiments.

### Overexpression of SimReg1

The codon-optimized copy of the *simReg1 *gene, named *simReg1s*, was synthesized by Mr. GENE Company (Heidelberg, Germany) and was provided on the plasmid pMA-simR1. Gene *simReg1s *was amplified from pMA-simR1 using primers SSR1F and SSR1R (Table 2). The PCR product was cloned into the pET21d NcoI-EcoRI sites, giving pETSR1c-15.

*E. coli *BL21(DE3) (pLysS) harboring the pETSR1c-15 plasmid was grown overnight at 37°C. LB (400 mL) containing 50 μg/mL of ampicillin was inoculated with 2 mL of the overnight culture and incubated at 21°C until the OD_600 nm _reached 0.7. SimReg1 expression was induced with 1 mM IPTG. After incubation for an additional 16 h, the cells were harvested by centrifugation and washed with ice-cold column buffer (20 mM Tris-HCl [pH 8.0], 50 mM NaCl). Cell lysis and purification of SimReg1 with His-tag-binding resins were performed according to Novagen instructions. SimReg1 was eluted with column buffer containing 200 mM imidazole. The purest fractions (as determined by SDS-PAGE and Coomassie blue staining) were pooled, washed with storage buffer (50 mM potassium phosphate [pH 8.0], 300 mM NaCl, 10% glycerol), concentrated using Amicon Ultra (Millipore). Aliquots of SimReg1 fusion protein in storage buffer were stored at - 80°C, or used immediately in DNA-binding assays.

### Electrophoretic mobility shift DNA-binding assays (EMSA)

DNA fragments containing putative promoters of *simD4 *(P_*D4*_, 513 bp), *simReg1 *(P_*R1*_, 490 bp), *simD3 *(P_*D3*_, 300 bp), *simX4 *(P_*X4*_, 350 bp), *simA7 *(P_*A7*_, 300 bp), *simEx2 *(P_*Ex2*_, 550 bp), *simB7 *(P_*SR3*_, 319 bp), *simX *(P_*SEx1*_, 280 bp), *simR *(P_*SR2*_, 300 bp), and the putative promoter region between *simX *and *simR *genes (P_*R2Ex*_, 780 bp) (Figure 2) were used in EMSA. Indicated promoter regions were amplified from the chromosomal DNA of *S. antibioticus *using primer pairs listed in Table 2. Each EMSA contained 50 ng of a target DNA and 0.9 μg, 1.8 μg, 2.7 μg, 3.6 μg, 4.5 μg of the His-SimReg1 protein in a total volume of 20 μL in a binding buffer (20 mM Tris HCl [pH 8.0], 1 mM EDTA, 1 mM DTT, 100 mM KCl, 1 mM MgCl_2_, 10% glycerol). After incubation for 25 min at room temperature, protein-bound and free DNA were separated by electrophoresis at 4°C on a 4.5% nondenaturing polyacrylamide gel in 0.5 × TBE buffer. The gel was stained with ethidium bromide and analyzed using a UV-imaging system (Fluorochem 5330). A negative control assay was carried out in the presence of the part of the *simD4 *coding region, amplified with the use of primers D4For and D4Rev (Table 2). Extracts from the strain *S. antibioticus *Tü6040 × pSsimR1-1, containing more then 95% of simocyclinones (Additional file 1), dissolved in methanol (5% and 10% - final volume in a reaction mixture) were tested as SimReg1 ligands.

## Results

### *In silico *analysis of the *simReg1 *gene product

**Figure 3:**
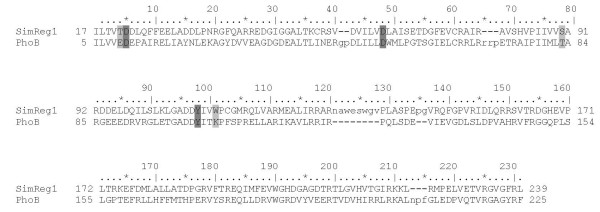
**Amino acid sequence comparison of the SimReg1 and PhoB (*E. coli*) proteins**. The conserved amino acids which are important for phosphorylation and consequence response are shaded in grey (aa that differ in proteins) and dark grey (aa that are identical in both sequences).

The putative product of the *simReg1 *gene is a 251 aa protein with a molecular mass 27.94 kDa. As evident from BLAST and CDD search results, putative amino acid sequence of the protein has significant similarity to response regulators in two component control systems. The closest homologues of SimReg1 are proteins that act as positive regulators for angucycline-like biosynthesis, including JadR1 from *S. venezuelae *(60% similarity) Wang et al. 2009, LanI from *S. cyanogenus *(58% similarity) Rebets et al. 2008 and LndI from *S. globisporus *(58% similarity) Rebets et al. 2003; Rebets et al. 2005. Analysis of the SimReg1 amino acid sequence using ExPASy Proteomics Server http://expasy.org revealed a putative signal receiver domain (the REC domain, aa 15-123) located at the N-terminal part of the protein and a DNA-binding domain at the C-terminus (aa 167-239). The latter is predicted to interact with short conserved regions of the target DNA and with the RNA polymerase. The secondary structure of the C-terminal DNA-binding domain of SimReg1 was similar to that of OmpR (*E. coli*) and PhoB (*E. coli*), which adopt a winged helix-turn-helix (HTH) moiety. In the REC domain of the regulatory protein PhoB, six conserved amino acid residues are believed to be vital for phosphorylation and consequence response Sola-Landa et al. 2003; Wang et al. 2009; Dyer and Dahlquist 2006, but only three of them are present in SimReg1 (Figure 3). Also, no protein kinase encoding genes have been found within the *sim *cluster. Thus, we suppose that SimReg1 belongs to "atypical" response regulators (ARR), like its close homolog JadR1 Wang et al. 2009.

### *S. antibioticus *ΔsimReg1 mutant is deficient in simocyclinone production

**Figure 4:**
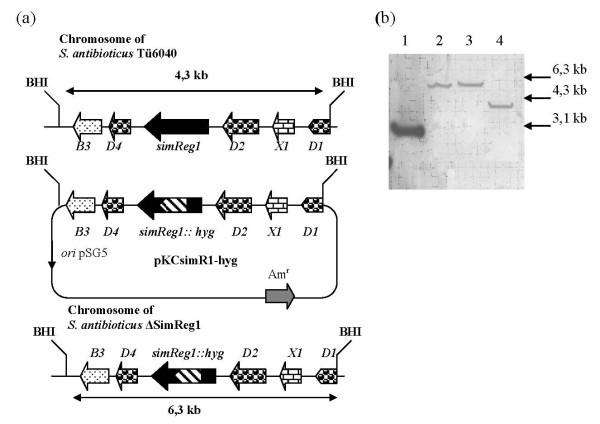
**Inactivation of the *simReg1 *gene**. (a) Schematic representation of the *simReg1 *gene disruption. (b) Results of the Southern hybridization of KpnI-digested plasmid pKCEsimR1 (1), BamHI digested total DNA samples from *S. antibioticus *ΔsimReg1 (2, 3) and Tü6040 (4) with 1.4 kb SmaI fragment harboring *simReg1 *gene.

**Figure 5:**
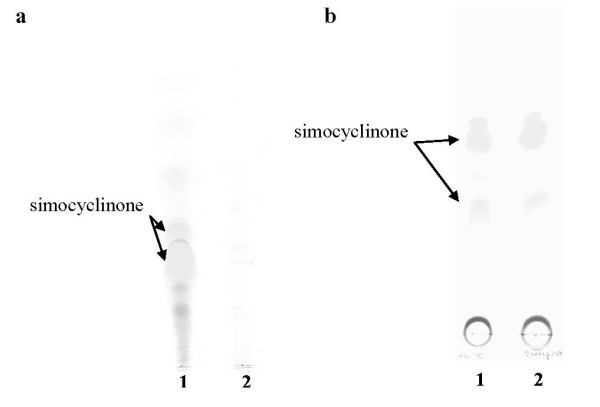
**TLC analysis of secondary metabolites produced by: (a) *S. antibioticus *Tü6040 (1), ΔsimReg1 (2) strains; (b) *S. antibioticus *Tü6040 (1), Tü6040 × pSSimR1-1 (2)**.

In order to investigate the function of *simReg1*, the chromosomal copy of the gene was replaced by the mutant allele containing a hygromycin resistance cassette (*hyg*) (Figure 4a). Inactivation of the *simReg1 *gene was proven by Southern hybridization. BamHI digested chromosomal DNA of the wild type and *S. antibioticus *ΔsimReg1 strains were probed with the DIG-labeled fragment containing *simReg1*, obtained as a KpnI fragment from the plasmid pKCEsimR1 Rebets et al. 2008. A single hybridization signal of the expected size (4.3 kb) was detected in the case of the wild type strain and a 6.3 kb fragment was detected in the ΔsimReg1 mutant (Figure 4b). The *S. antibioticus *ΔsimReg1 mutant had growth and morphological characteristics identical to those of the wild type. HPLC and TLC analysis (Figure 5a) of the extracts from the mutant strain ΔsimReg1 revealed no simocyclinone and its precursors, indicating that this gene is essential for antibiotic production.

To exclude any possibility of polar effects and to confirm that the cessation of simocyclinone production was caused by the inactivation of the *simReg1*, complementation experiment was carried. For this purpose, we used the pSSimR1-1 plasmid (Table 1), which contains the *simReg1 *gene under its own promoter cloned in the integrative vector pSET152. This plasmid was transferred into *S. antibioticus *wild type strain by means of conjugation. The recombinant strain *S. antibioticus *ΔsimReg1 × pSSimR1-1 was found to accumulate simocyclinone at a level comparable to those of the wild type (Figure 5b).

It is known that very often overexpression of the positive pathway-specific regulators lead to overproduction of antibiotics (Bibb 2005; Novakova et al., 2011). To analyze the effect of additional copies of *simReg1 *gene on simocyclinone biosynthesis, we introduced the plasmid pSsimR1-1 that contains *simReg1 *gene under its own promoter, into the wild type strain. Recombinant strain *S. antibioticus *Tü6040 × pSSimR1-1 produced in average 2.5 times more simocyclinone then the wild type.

### SimReg1 binds to the putative promoter regions of structural, transporter genes and its own gene

**Figure 6:**
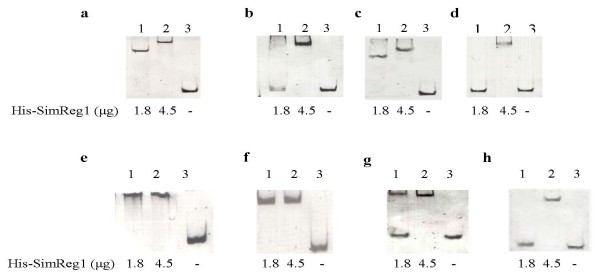
**Results of an EMSA performed to detect interactions of His-SimReg1 to promoter regions of the *sim *cluster**. In "a" promoter P_*D4 *_was used, in "b" P_*R1*_, in "c" P_*D3*_, in "d" P_*X4*_, in "e" P_*R2Ex*_, in "f" P_*A7*_, in "g" P_*Ex2*_, and in "h" P_*SR3*_.

In order to prove the DNA binding activity of SimReg1, gel mobility-shift assays were carried out. His-SimReg1 was purified (Additional file 2) and an *in vitro *binding assay was performed using His-SimReg1 and DNA fragments containing putative promoters of the regulator gene *simReg1 *(P_*R1*_), the 3-keto-acyl-reductase gene *simD4 *(P_*D4*_), the oxygenase gene *simA7 *(P_*A7*_), the transporter gene *simEx2 *(P_*Ex2*_), the 3-keto-acyl-reductase gene *simD3 *(P_*D3*_), the putative gene *simX4 *(P_*X4*_), the putative olivosyltransferase gene *simB7 *(P_*SR3*_), and the intergenic region between *simR *and the transporter gene *simEx1 *(hereafter *simX*) (P_*R2Ex*_) (Figure 2). Shifted bands were detected using the promoter regions of the enzyme encoding genes (Figure 6a, c, d, f), the transporter gene *simEx2 *(Figure 6g) and the regulatory gene *simReg3*, which is likely co-transcribed with the genes *simB7, simB5, simB4, simX5 *and *simX7 *(Figure 6h). Furthermore, DNA retardation occurred (Figure 6b) when the promoter of the *simReg1 *gene was used in the binding assay, indicating that SimReg1 is an autoregulatory protein. We carried out a set of control assays to demonstrate the specificity of the SimReg1 binding. For instance, none of the compounds in the crude extract of *E. coli *BL21(DE3) binds to any of the putative promoters (data not shown). We also showed that randomly chosen DNA did not interact with SimReg1 (Additional file 3).

**Figure 7:**
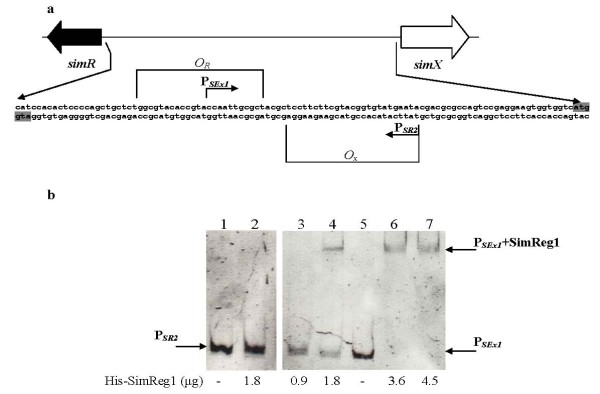
**Results of EMSA performed to detect interactions of His-SimReg1 to P_*SR2 *_and P_*SEx1 *_**. (a) Schematic representation of the intergenic region between *simR *and *simX*. Operators *O_X _*and *O_R _*are also shown (according to Le et al. 2009). Translation start codons are highlighted in dark grey. P_*SR2 *_and P_*SEx1 *_- indicate putative promoter regions used in EMSA. (b) Lane 1: P_*SR2*_; lane 2: P_*SR2 *_+ His-SimReg1; lane 3: P_*SEx1*_; lane 4: P_*SEx1 *_+ His-SimReg1.

SimReg1 was found to bind to the DNA fragment containing the *simR*/*simX *intergenic region (Figure 6e). However, it was not known whether SimReg1 interacts with the promoters of both genes. A 67 bp fragment located in front of the start codon of *simR *(P_*SR2*_) and a 69 bp fragment located in front of *simX *(P_*SEx1*_) (Figure 7a) were used for additional EMSA analysis. No binding was identified with the P_*SR2 *_promoter, whereas DNA retardation occurred when the P_*SEx1 *_promoter was used in the assay (Figure 7b). These results indicate that SimReg1 is capable of binding to the promoter region of *simX*.

### Effect of culture extracts from *S. antibioticus *Tü6040 × pSSimR1-1 on the activity of SimReg1

**Figure 8:**
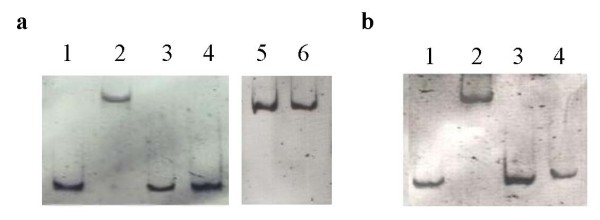
**Results of an EMSA performed to investigate the influence of crude extracts from *S. antibioticus ***ü6040 × pSSimR1-1 strain on the interactions of SimReg1 to promoter regions of the *sim *cluster. In "a" promoter P_*R1 *_and in "b" P_*A7 *_were used. (a) lane 1: P_*R1*_; lane 2: P_*R1 *_+ His-SimReg1; lane 3: P_*R1 *_+ His-SimReg1 + crude extract isolated from *S. antibioticus *Tü6040 *× *pSSimR1-1 (5% of total reaction volume); lane 4: P_*R1 *_+ His-SimReg1 + crude extract isolated from *S. antibioticus *Tü6040 × pSSimR1-1 (10% of total reaction volume); lane 5: P_*R1 *_+ His-SimReg1 + methanol (5% of total reaction volume); lane 6: P_*R1 *_+ His-SimReg1 + methanol (10% of total reaction volume); (b) lane 1: P_*A7*_; lane 2: P_*A7 *_+ His-SimReg1; lane 3: P_*A7 *_+ His-SimReg1 + crude extract isolated from *S. antibioticus *Tü6040 × pSSimR1-1 (5% of total reaction volume); lane 4: P_*A7 *_+ His-SimReg1 + crude extract isolated from *S. antibioticus *Tü6040 × pSSimR1-1 (10% of total reaction volume)

Since DNA binding ability of JadR1, which also belongs to ARR and is very similar to SimReg1 (60% similarity), is regulated by jadomycin B Wang et al. 2009, we studied the effects of simocyclinone extracts from the *S. antibioticus *Tü6040 *× *pSSimR1-1 on the DNA binding activity of SimReg1. For this purpose the culture broth of *S. antibioticus *Tü6040 *× *pSSimR1 strain grown for 72 hours was extracted with an ethyl acetate, dried and dissolved in methanol. In overall the percentage of different types of simocyclinone in such an extract was more than 95% (Additional file 1). Presence of these extracts could dissociate His-SimReg1 from the promoter regions P_*R1 *_and P_*A7*_, as a result no shifted bands occurred (Figure 8). This effect was not due to methanol, the simocyclinone D8 solvent, as equivalent amounts of methanol had no effect on His-SimReg1-DNA complex formation (Figure 8).

### SimReg1 activates expression of a *gusA *reporter gene from P_*D4 *_promoter

**Figure 9:**
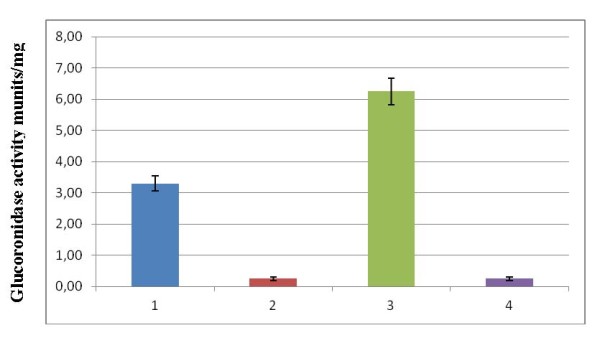
**Glucuronidase activity in cell lysates of *S. lividans ***strains: 1 - *S. lividans*×pSimD4script; 2 - *S. lividans*×pGUS; 3 - *S. lividans*×pSimD4script/pUWLsimReg1; 4 - *S. lividans*×pGUS/pUWLsimReg1.

On the basis of the gene inactivation, overexpression and EMSA results we suppose that SimReg1 is a positive regulator of simocyclinone production. To investigate whether SimReg1 can activate the expression of the structural genes under heterologous conditions, a reporter system on the basis of *gusA *was used. For these purpose, we constructed two plasmids pSimD4script and pUWLsimReg1 (Table 1). In the first plasmid the promoter region of the putative ketoreductase gene *simD4 *(P_*D4*_) was fused with the coding sequence of the *gusA *gene. As a result expression of the reporter *gusA *is controlled by P_*D4*_. In the plasmid pUWLsimReg1 intact gene *simReg1 *was cloned under the control of erythromycin resistance gene promoter to make the expression of the regulatory gene constitutive. As it is evident from the EMSA analysis SimReg1 binds to the promoter of the gene *simD4 *(Figure 6a) this means that SimReg1 should influence expression from this promoter. To verify this assumption, both plasmids were transferred into heterologous host *S. lividans *1326 to avoid influence of two other regulatory proteins SimR and SimReg3 Trefzer et al., 2002. We obtained two strains: *S. lividans *harboring only pSimD4script and *S. lividans *harboring both plasmids pSimD4script and pUWLsimReg1. As a negative control we used strains: *S. lividans *1326 *× *pGUS to show that there is no GusA activity when *gusA *gene contains no promoter and *S. lividans *1326 harboring both plasmids pGUS (Table 1) and pUWLsimReg1 to demonstrate that SimReg1 specifically binds only to *simD4 *promoter region and that SimReg1 can't influence *gusA *expression in the absence of this promoter. Aforementioned four strains were grown in liquid TSB medium for 5 days and samples of the strains were used for GusA activity measurement as described in Materials and Methods. In the control strains the activity of GusA was approximately 0.25 ± 0.06 (Figure 9). In the case of the *S. lividans *strain that contains *gusA *gene under P_*D4 *_activity was in average 3.3 ± 0.24 (Figure 9). In the strain containing both *gusA *gene under P_*D4 *_promoter and the SimReg1 protein the activity was 6.25 ± 0.43 (Figure 9). It is in overall two times higher than without the protein. On the basis of these results, we may conclude that SimReg1 binds to the *simD4 *promoter region.

## Discussion

Simocyclinone is a potent antibacterial drug that inhibits DNA gyrase supercoiling Oppegard et al. 2009; Sadig et al. 2010; Edwards et al. 2009; Sissi et al. 2009. The gene cluster responsible for simocyclinone production was cloned and biosynthetic, and regulatory genes were detected Trefzer et al. 2002; Galm et al. 2002. Here, we report on the function of the gene *simReg1 *involved in the regulation of simocyclinone production and export.

SimReg1, to our knowledge, is the first OmpR-PhoB subfamily regulator identified within aminoucoumarin biosynthetic gene clusters. It appears to be a key regulator of simocyclinone production since inactivation of *simReg1 *completely abolished antibiotic biosynthesis and its overexpression in the wild type strain *S. antibioticus *Tü6040 led to almost 2.5 times increase in simocyclinone production. *In silico *analysis and DNA shift assays showed that SimReg1 is a DNA-binding autoregulatory protein that interacts directly with putative promoter regions of the structural *sim *genes, both transporter genes *simX *and *simEx2*, and the putative regulatory gene *simReg3*. Our results indicate that SimReg1 is an activator of the structural and transporter genes transcription, as expression of the reporter gene *gusA *under P_*D4 *_in the presence of SimReg1 was at least two times higher, than without it. DNA-binding activity of SimReg1 is abolished in the presence of extracts from *S. antibioticus *Tü6040 *× *pSSimR1-1. As extracts used in the experiment were enriched with simocyclinones, these might indicate the existence of autoregulation by binding most likely simocyclinone or its intermediates. However to establish this assumption additional experiments are required. Similar autoregulation by binding of the end product was described for JadR1 Wang et al. 2009, the close homolog of SimReg1. An interesting finding is that SimReg1 binds to the promoter region of the exporter gene *simX*. SimR is known to repress expression of *simX *and its own gene by binding to two distinct operators within the *simR*/*simX *intergenic region Le et al. 2009. SimR was shown to dissociate from the *simX *promoter in the presence of simocylinone D8 Le et al. 2009; Le et al. 2011a; Le et al. 2011b. At the same time SimReg1 is interacting with the 69 bp DNA region upstream to the start codon of *simX*. This means that the operator of SimReg2 partially overlaps with the DNA-binding region of SimReg1. Therefore, it is very likely that in the presence of simocyclinone dissociation of SimReg2 from the promoter region of *simX *is necessary for SimReg1 binding indicating that SimReg1 and SimReg2 compete for the binding to the *simX *promoter.

The presence of distinct regulatory proteins indicates the importance for the cell to strongly control simocyclinone production and transport. The structure of simocyclinone is assembled from products of three distinct biosynthetic routes. To produce such a complex molecule the biosynthetic pathway and the transport have to be precisely tuned and controlled.

Based on our data and the data described by Buttner and coworkers Le et al. 2009; Le et al. 2011a; Le et al. 2011b, we proposed the following preliminary model for the regulation of simocyclinone biosynthesis and export. When the concentration of simocyclinone and/or its intermediates is low the transcription of the exporter gene *simX *is repressed by SimR. At the same time, SimReg1, being the key regulator of simocyclinone biosynthesis, activates expression of the structural *sim*-genes and simocyclinone production. When the cellular concentration of simocyclinone exceeds a certain level, SimR is released from P_*SEx1 *_that allows SimReg1 to bind to the promoter. This activates *simX *expression, followed by the transport of simocyclinones out of the cell. This mechanism couples the biosynthesis of simocyclinone to its export. In such a way, an additional mechanism of exact tuning of biosynthesis level is exerted ensuring the protection of the producing bacteria from the toxicity of its secondary metabolism product.

The present study portrays a strong link between antibiotic production and export and describes for the first time the function of the atypical response regulator in the control of the biosynthesis of simocyclinone. Furthermore, our data suggest a useful biotechnological approach for optimization of simocyclinone production, as overexpression the gene encoding positive regulator SimReg1 leads to antibiotic overproduction.

## Competing interests

The authors declare that they have no competing interests.

## Supplementary Material

Additional file 1 **HPLC analysis of secondary metabolites produced by *S. antibioticus *Tü6040 × pSSimR1-1**. On axis *y *relative absorption units (AU) are plotted. On axis *x *retention time of compounds is plotted (in min). Under conditions stated SD8 has *R_t _*of 24.7 min. The overall content of simocyclinones in the extract was around of 95%. Click here for file

Additional file 2 **Purification of the His-tagged SimReg1 protein from *E. coli *BL21 (DE3)**. Lane 1: molecular mass marker (Pierce Protein Research Products); lane 2: flow through; lane 3: purified SimReg1. Click here for file

Additional file 3 **Results of EMSA performed to detect interactions of SimReg1 to part of the *simD4 *gene**. Lane 1: *simD4*; lane 2: *simD4 *+ His-SimReg1; lane 3: *simD4 *+ His-SimReg1; lane 4: *simD4 *+ His-SimReg1. Click here for file
