# Supplementary figures and images for: SimReg1 is a master switch for biosynthesis and export of simocyclinone D8 and its precursors
Source: AMB Express. 2012 Jan 3;2:1. doi: 10.1186/2191-0855-2-1 (PMC3261101; doi:10.1186/2191-0855-2-1)

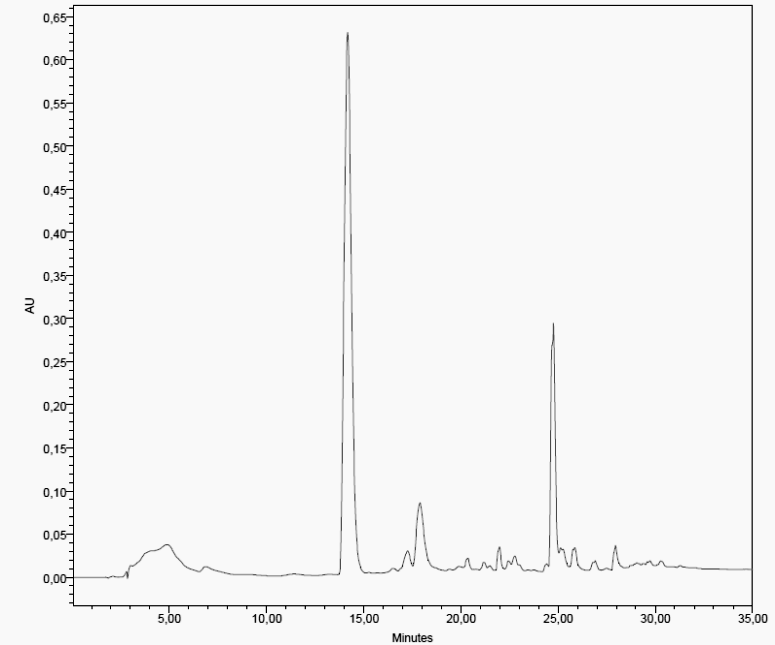


simD

simC

simD

simA

simB

Supplement: Additional file 1 — HPLC analysis of secondary metabolites produced by S. antibioticus Tü6040 × pSSimR1-1. On axis y relative absorption units (AU) are plotted. On axis x retention time of compounds is plotted (in min). Under conditions stated SD8 has Rt of 24.7 min. The overall content of simocyclinones in the extract was around of 95%. [file 2191-0855-2-1-S1.DOC]

1 2 3

18.4 kDa

14.4 kDa

25.0 kDa

45.0 kDa

66.2 kDa


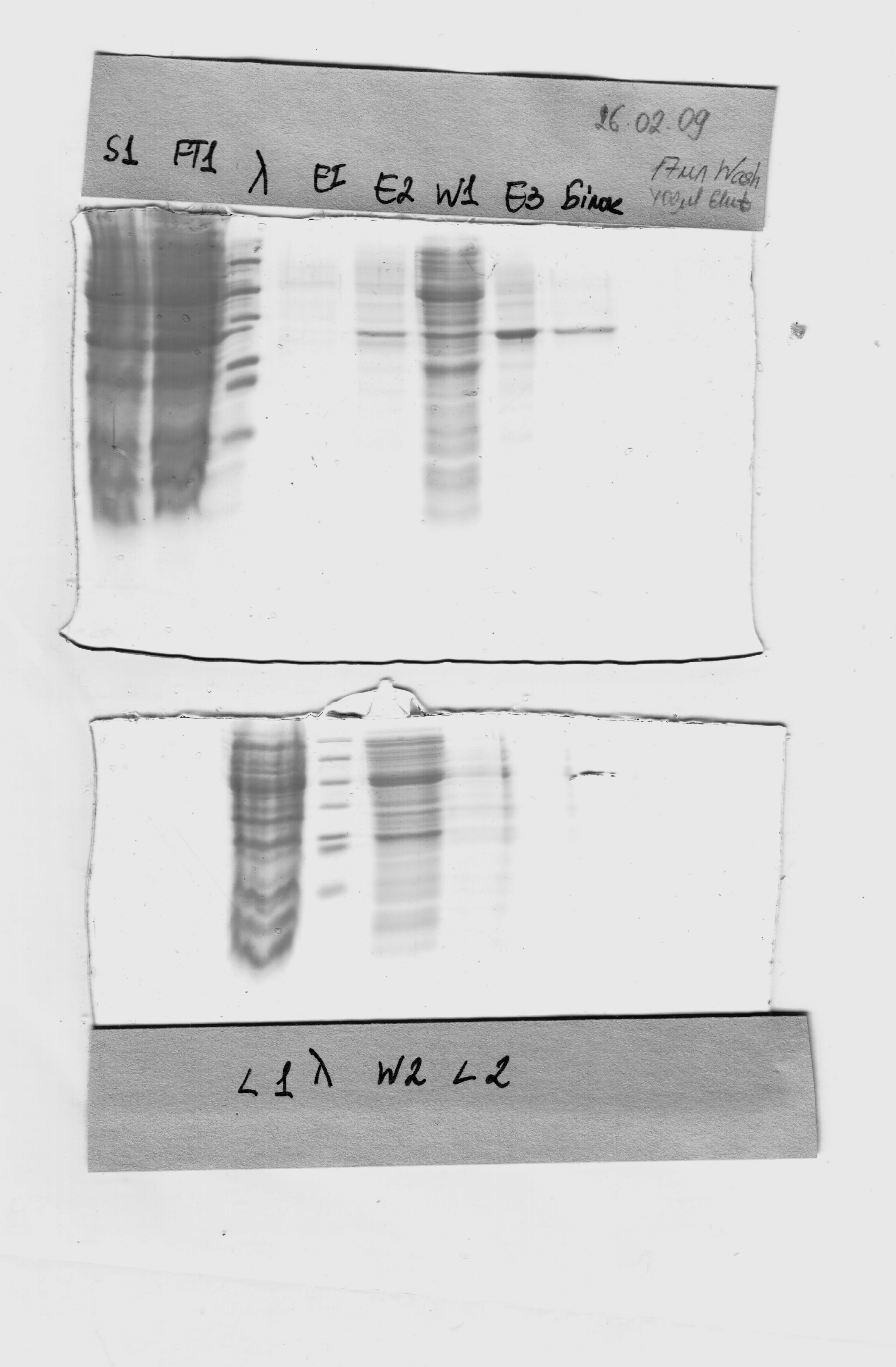

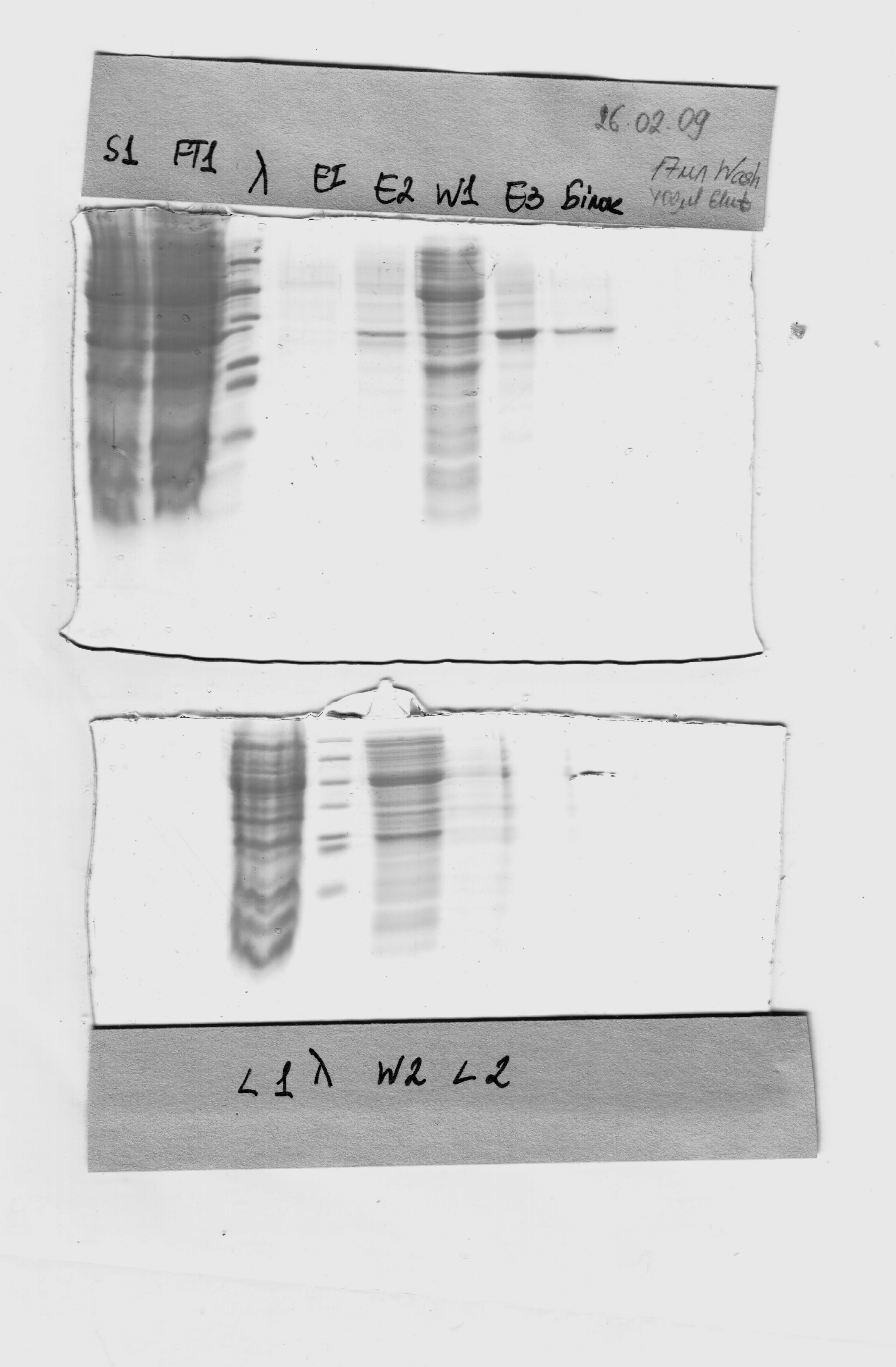


SimReg1

35.0 kDa

116.0 kDa


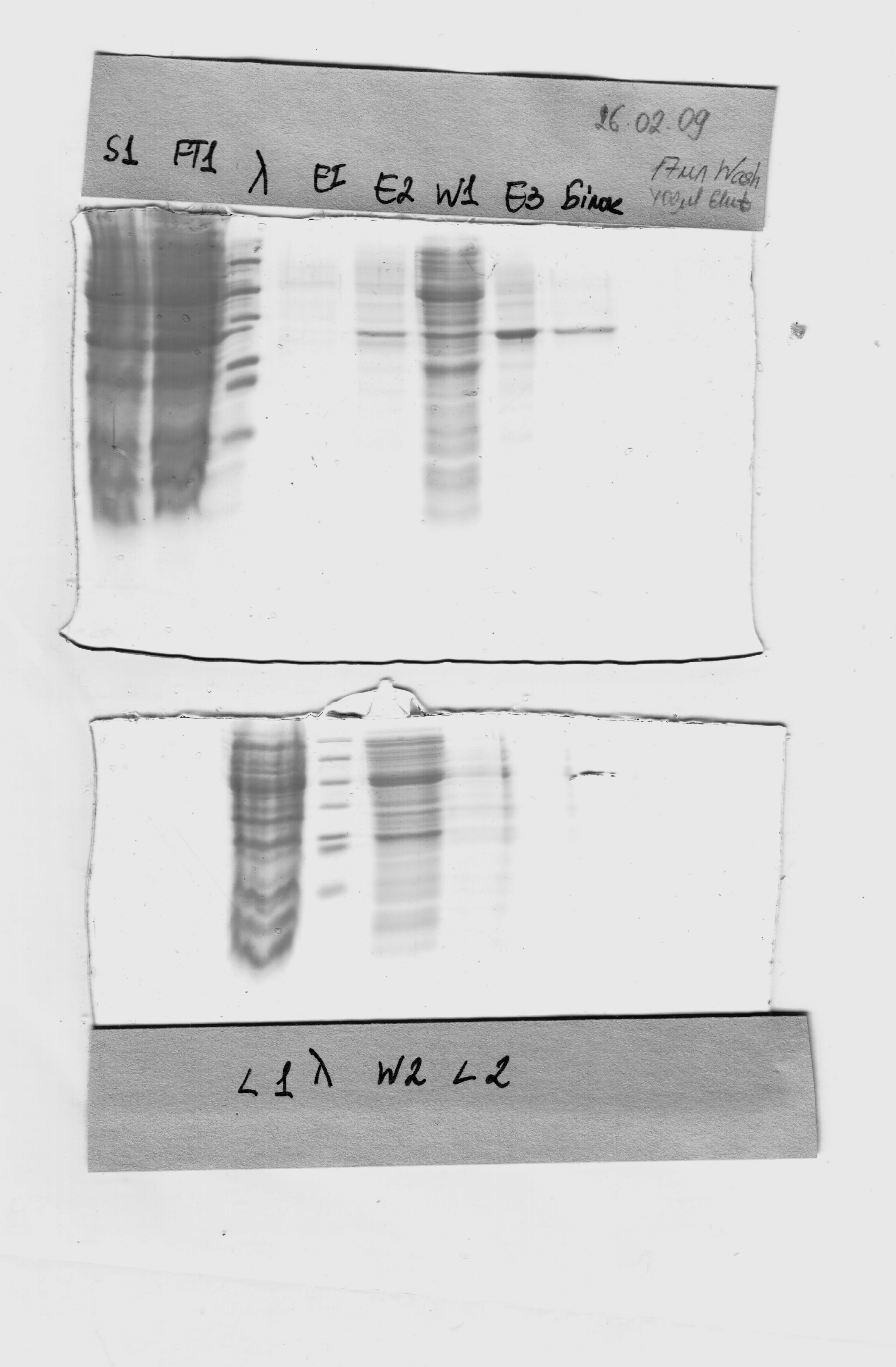

Supplement: Additional file 2 — Purification of the His-tagged SimReg1 protein from E. coli BL21 (DE3). Lane 1: molecular mass marker (Pierce Protein Research Products); lane 2: flow through; lane 3: purified SimReg1. [file 2191-0855-2-1-S2.DOC]

1 2 3 4


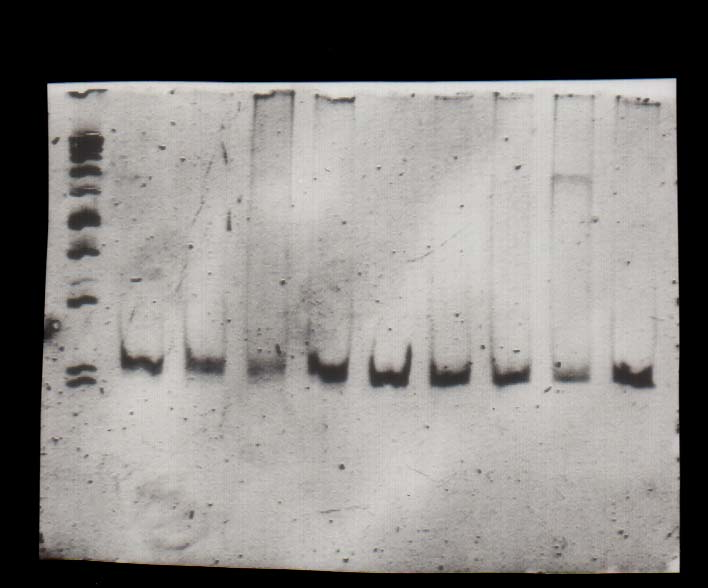


*simD4*

SimReg1 (μg) - 0.9 1.8 4.5

Supplement: Additional file 3 — Results of EMSA performed to detect interactions of SimReg1 to part of the simD4 gene. Lane 1: simD4; lane 2: simD4 + His-SimReg1; lane 3: simD4 + His-SimReg1; lane 4: simD4 + His-SimReg1. [file 2191-0855-2-1-S3.DOC]
